# A SH3_5 Cell Anchoring Domain for Non-recombinant Surface Display on Lactic Acid Bacteria

**DOI:** 10.3389/fbioe.2020.614498

**Published:** 2021-01-27

**Authors:** Pei Kun Richie Tay, Pei Yu Lim, Dave Siak-Wei Ow

**Affiliations:** Microbial Cells Group, Bioprocessing Technology Institute, Agency for Science, Technology and Research (A^*^STAR), Singapore, Singapore

**Keywords:** lactic acid bacteria, cell anchoring domain, bacteria surface display, bacteria protein delivery, superoxide dismutase, probiotics

## Abstract

Lactic acid bacteria (LAB) are a group of gut commensals increasingly recognized for their potential to deliver bioactive molecules *in vivo*. The delivery of therapeutic proteins, in particular, can be achieved by anchoring them to the bacterial surface, and various anchoring domains have been described for this application. Here, we investigated a new cell anchoring domain (CAD4a) isolated from a Lactobacillus protein, containing repeats of a SH3_5 motif that binds non-covalently to peptidoglycan in the LAB cell wall. Using a fluorescent reporter, we showed that C-terminal CAD4a bound *Lactobacillus fermentum* selectively out of a panel of LAB strains, and cell anchoring was uniform across the cell surface. Conditions affecting CAD4a anchoring were studied, including temperature, pH, salt concentration, and bacterial growth phase. Quantitative analysis showed that CAD4a allowed display of 10^5^ molecules of monomeric protein per cell. We demonstrated the surface display of a functional protein with superoxide dismutase (SOD), an antioxidant enzyme potentially useful for treating gut inflammation. SOD displayed on cells could be protected from gastric digestion using a polymer matrix. Taken together, our results show the feasibility of using CAD4a as a novel cell anchor for protein surface display on LAB.

## Introduction

Microbial cell-surface display has a wide range of biotechnological and industrial applications. It can be used to screen protein and peptide libraries in directed evolution, epitope mapping and drug discovery (Rockberg et al., 2008; Hudson et al., 2012; Fleetwood et al., 2013; Robert and Gouet, 2014). Microbes displaying proteins are also useful as remedial biosorbents (Tang et al., 2014; Hui et al., 2018; Maruthamuthu et al., 2018), biosensors (Han et al., 2018; Park, 2020), whole-cell biocatalysts (Pontes et al., 2012), and as vaccines and delivery vectors for therapeutics (Cano-Garrido et al., 2015; Plavec and Berlec, 2019). Lactic acid bacteria (LAB) are a heterogeneous group of Gram-positive bacteria, commonly of the genera *Lactococcus, Lactobacillus, Streptococcus, Pediococcus*, and *Leuconostoc*. They have a long history as components of fermented foods and are thus considered GRAS (“generally regarded as safe” per U.S. Food and Drug Administration). They are used industrially in feed and food fermentation, and in the production of various fine chemicals (Mora-Villalobos et al., 2020). Many lactobacilli also colonize mucosa in humans and animals, forming part of the intestinal and vaginal microbiomes, and probiotic strains of *Lactobacillus* have been identified that confer health benefits to the host (Gill and Prasad, 2008; Walter, 2008). These characteristics make LAB valuable candidates for protein display in numerous industrial and biomedical applications.

LAB displaying enzymes can be used as biocatalysts for industrial processes. Nguyen et al. displayed β-mannanase on the surface of *Lactobacillus plantarum* for the production of manno-oligosaccharides, a class of prebiotic oligosaccharides (Nguyen et al., 2019). Similarly, Pham et al. displayed dimeric β-galactosidases on *L. plantarum* for lactose conversion and production of galacto-oligosaccharides (Pham et al., 2019). In both cases, the bacterial catalysts could be used for multiple rounds of bioconversion. Other groups displayed cohesins on *L. plantarum* and *Lactococcus lactis* to assemble multi-enzyme cellulosomal complexes for the degradation of complex polymers (Wieczorek and Martin, 2012; Stern et al., 2018). Surface display could also be used to introduce substrate-binding domains on LAB to enable cell immobilization on solid supports for continuous bioprocessing, as has been demonstrated for *L. lactis* displaying a chitin-binding domain (Simşek, 2014).

LAB have also been investigated for therapeutic use, for instance, to treat metabolic and gastrointestinal diseases. Companies like Aurealis Pharma and Precigen ActoBio are developing “live biotherapeutics” using engineered strains of *Lactococcus lactis* that secrete therapeutic proteins and peptides in the oral and gastrointestinal tract. Anchoring these therapeutic entities to the bacteria surface could provide protection against proteolysis during gastrointestinal transit (Mao et al., 2016). Proteins that have been successfully displayed on *L. lactis* include β-galactosidase to manage lactose intolerance; an insulin analog (SCI-59) to manage diabetes; and the thrombolytic agent subtilisin QK-2 (Mao et al., 2016, 2017; Yin et al., 2018). Various protein domains that block pro-inflammatory cytokines and chemokines have been also displayed on LAB to treat inflammatory bowel disease (IBD) (Kosler et al., 2017; Škrlec et al., 2017; Plavec et al., 2019). Škrlec et al. displayed a pentadecapeptide BPC-157 to reduce the production of reactive oxygen species (ROS) to moderate gut inflammation (Skrlec et al., 2018). Protein display on LAB can also be used to develop bacterial vaccine vectors. Here the innate immunogenicity of certain probiotic strains may obviate the need for adjuvants. Lactic acid bacteria engineered to display antigens from influenza A, pneumococcus, *Mycobacterium tuberculosis*, and SARS-CoV have shown efficacy as mucosal vaccines against their respective viral and bacterial pathogens in animal models (Lee et al., 2006; Hernani Mde et al., 2011; Chowdhury et al., 2014; Mustafa et al., 2018). LAB can also be developed into efficient vectors for DNA delivery through the surface display of targeting proteins that directly interact with host epithelial or immune cells (Pontes et al., 2012; Liu et al., 2019).

To enable surface display, a protein or peptide is fused to an anchoring domain that binds to the LAB cell wall. Such anchoring domains may be covalently linked to a cell wall component, or they may bind non-covalently. Examples of covalent anchors include lipoproteins like BmpA and PrsA (Fredriksen et al., 2012; Zadravec et al., 2014); transmembrane proteins like PgsA (Narita et al., 2006; Lei et al., 2011); and more commonly, LPXTG domains derived from the streptococcal M6 protein or the *L. plantarum* Lp_2578 protein, which are anchored to peptidoglycans by cell wall sortases (Dieye et al., 2001; Fredriksen et al., 2010). Bacteria are usually genetically modified for covalent display, but the use of GM bacteria raises safety concerns and may encounter lower consumer acceptance and more severe regulatory scrutiny, especially when used in food or pharmaceutical preparations. Non-covalent anchoring strategies avoid the use of recombinant bacteria as hosts for protein display. Proteins containing non-covalent anchoring domains can be produced in an expression strain, then anchored *in trans* to a wild-type (non-GM) host LAB strain. Another advantage of this approach is that protein production is not limited by the biosynthetic capabilities of the host bacterium, and can undergo further post-translational modifications prior to surface anchoring.

The success of a non-covalent cell surface display system depends on choosing an appropriate anchoring motif for the target protein and host cell. Each anchoring domain has a different capacity for protein display, and can be highly selective of its target LAB. While several non-covalent binding domains have been identified, only a few have been applied for protein surface display. These include: lactobacillal S-layer homology domains (Åvall-Jääskeläinen et al., 2002; Hu et al., 2011); WxL (Brinster et al., 2007), SH3 (Plavec et al., 2019); CW_1 (Plavec et al., 2019) and LysM (Raha et al., 2005; Hu et al., 2010; Ravnikar et al., 2010; Xu et al., 2011). Although many of these domains have known binding partners, the mechanism of binding is still ambiguous for some (Desvaux et al., 2018). The LysM domain from the lactococcal protein AcmA is a commonly used non-covalent anchor for LAB surface display (Steen et al., 2005; Bosma et al., 2006; Ravnikar et al., 2010). New anchoring motifs are constantly being sought to target a wide range of LAB, and to allow surface display of different proteins on the same cell. The bacterial SH3 type 5 motif (SH3_5; Pfam PF08460) is known to bind cell wall peptidoglycans in Gram-positive bacteria (Becker et al., 2009; Mitkowski et al., 2019). It contains 60–65 amino acids and is mainly found among Firmicutes, especially of the *Streptococcus* and *Lactobacillus* genera (Desvaux et al., 2018). A recent report described the use of a lactococcal phage SH3_5 motif for surface display in *L. lactis* (Plavec et al., 2019). Nonetheless, SH3_5 has not been widely investigated for bacterial protein display. The goal of the present study was to test a newly-identified anchoring domain (CAD4a) containing SH3_5 repeats for heterologous protein display on LAB. The CAD4a domain was isolated from an *L. plantarum* protein. We appended this domain to two proteins—a fluorescent reporter and a dimeric enzyme—and examined functional display on LAB, as well as conditions for optimal anchoring, and resistance of the anchored proteins to gastric digestion.

## Materials and Methods

### Bacterial Strains, Culture Conditions, and Plasmid Assembly

The bacteria strains and plasmids used in this study are listed in Table 1. Cloning was performed in *E. coli* Turbo and proteins were expressed in *E. coli* BL21(DE3) as detailed in the next section. *E. coli* was selected on LB agar supplemented with 100 μg/ml carbenicillin. Lactic acid bacteria were grown in static, unaerated MRS broth (Sigma-Aldrich) at 37°C.

**Table 1:** Bacterial strains and plasmids used in this study.

	**Feature**	**Source**
**Strains**		
*E. coli* Turbo	Cloning host, TG1 derivative *glnV44 thi-1* Δ*(lac-proAB) galE15 galK16* *R*(*zgb-210*::Tn*10*)Tet^S^ *endA1 fhuA2* Δ*(mcrB-hsdSM)5*, (*r_*K*_*^−^*m_*K*_*^−^) F′[*traD36 proAB*^+^ *lacI*^q^ *lacZΔM15*]	NEB
*E. coli* BL21(DE3)	Expression host, *E. coli* str. B F^−^*ompT gal dcm lon hsdS_*B*_*(*r_*B*_*^−^*m_*B*_*^−^) λ(DE3 [*lacI lacUV5*-*T7p07 ind1 sam7 nin5*]) [*malB*^+^]_K−12_(λ^S^)	Thermo Fisher
*Lactococcus lactis* NZ9000	Binding host, MG1363 derivative *pepN*::*nisRK*	MoBiTec
*Lactobacillus casei* 393	Binding host, wild type	ATCC
*Lactobacillus fermentum* 14931	Binding host, wild type	ATCC
*Lactobacillus plantarum* 8014	Binding host, wild type	ATCC
*Lactobacillus rhamnosus* GG	Binding host, wild type	Lesaffre
**Plasmids**		
pET22b	P_T7_, Amp^R^, *lacI* gene, N-terminal pelB seq	Novagen
pET22b-Sirius	His-tagged Sirius	This study
pET22b-Sirius-CAD4a12	12-residue spacer between Sirius and CAD4a	This study
pET22b-Sirius-CAD4a24	24-residue spacer between Sirius and CAD4a	This study
pET22b-Sirius-CAD4a36	36-residue spacer between Sirius and CAD4a	This study
pET22b-SOD	His-tagged SOD	This study
pET22b-SOD-CAD4a12	12-residue spacer between SOD and CAD4a	This study
pET22b-SOD-CAD4a24	24-residue spacer between SOD and CAD4a	This study
pET22b-SOD-CAD4a36	36-residue spacer between SOD and CAD4a	This study

Supplementary Table 1 lists the primers and synthetic gene fragments used in this study. Primers and gene fragments were synthesized by Integrated DNA Technologies (USA). Gibson assembly was used to construct all plasmids. The pET22b plasmid was linearized with primers F1 and R1, and assembled with fragment G10 to give pET22b-Sirius. pET22b-Sirius was linearized with primers F1 and either R7 or R6, to give pET22b-Si-CAD4a12 and -Si-CAD4a24, respectively, after assembly with gene fragment G5. CAD4a was subcloned from pET22b-Si-CAD4a24 using primers F15 and R5, then assembled with pET-Sirius linearized with F1 and R26, to give pET22b-Si-CAD4a36.

For the SOD constructs, G29 was amplified with primers F18 and R37, and pET22b-Sirius linearized with primers F1 and R4. Both fragments were then assembled to give pET22b-SOD. pET22b-SOD was linearized with primers F1 and either R24 or R25, to give pET22b-SOD-CAD4a12 and -SOD-CAD4a24, respectively, after assembly with gene fragment G5. CAD4a was subcloned from pET22b-Si-CAD4a24 with primers F15 and R5, then assembled with pET-SOD linearized with F1 and R27, to give pET22b-SOD-CAD4a36.

### Protein Expression

Overnight *E. coli* BL21(DE3) cultures were diluted 1:100 in Terrific Broth and grown to optical density OD_600_ ~0.8. At that point, the temperature was reduced to 20°C and sorbitol was added to a concentration of 0.4 M. Sorbitol was added only for expression of CAD4a protein conjugates, to reduce protein aggregation. Expression was induced with 0.2 mM IPTG and allowed to proceed for 6 h at 20°C. Cells were then pelleted at 4,000 g for 10 min, resuspended in Tris buffer (50 mM Tris, 0.3 M NaCl, pH 8), and subjected to one freeze-thaw cycle before lysis on ice with a probe sonicator (Microson XL2000, 10 s ON, 10 s OFF, 8 cycles). The lysate was pelleted at 12,000 g for 30 min at 4°C, and separated on Ni-NTA resin (Qiagen, USA) in a PD-10 column. His-tagged protein was eluted with 200 mM imidazole, then concentrated and buffer-exchanged into 1 × PBS (pH 7.4) and stored at 4°C until use.

### SDS-PAGE and Western Blot

Protein concentrations were determined using Bradford reagent (Biorad). Protein samples were analyzed on NuPAGE 4–12% Bis-Tris gels (Life Technologies), following manufacturer's protocols. Gels were stained with InstantBlue (Expedeon) or transferred onto nitrocellulose membranes using the semi-dry method at 20 V for 20 min (Trans-Blot, Bio-Rad). The membrane was washed with TBST (1 × TBS, 0.1% Tween 20), blocked with 5% w/v non-fat dry milk in TBST for 1 h at room temperature, then exposed to a 1:10,000 dilution of HRP-conjugated anti-His antibody (Merck) for 1 h at room temperature before detection with Clarity Western ECL Blotting Substrate (Bio-Rad) using the manufacturer's protocol. Gel images were acquired on a ChemiDoc MP imaging system (Bio-Rad), and blots were imaged on an ImageQuant LAS 500 imager (GE Healthcare).

### Anchoring of Sirius-CAD4a to Lactic Acid Bacteria

*Lactobacillus casei, L. fermentum, L. plantarum, L. rhamnosus*, and *Lactococcus lactis* were grown to mid-log (OD_600_ 0.8–1.2) or stationary phase (overnight culture), washed once with 10% glycerol, then resuspended in a 50:50 mix of MRS and 20% glycerol, aliquoted, and frozen at −80°C to obtain stocks for subsequent cell binding studies. Except where stated otherwise, the following protocol was used for binding studies. Frozen log-phase cells were thawed and washed twice with binding buffer (1×PBS, pH 5), diluted to OD_600_ = 1.5, and resuspended in 75 μl of binding buffer containing 2 μM of Sirius-CAD4a12. The mixture was incubated for 1.5 h at 37°C with periodic mixing, then pelleted and washed twice with the same binding buffer before transfer to a black 96-well-polystyrene plate for fluorescence measurement. Cell-associated fluorescence was measured on a spectrophotometer (Tecan, USA) with excitation at 355 nm and emission at 424 nm. The background fluorescence of the cells was subtracted to obtain a reading in relative fluorescence units (RFU). All studies were performed in triplicate. Cell imaging was performed on a Nikon Eclipse Ni-U microscope using a DAPI filter and 60× oil immersion lens.

### Influence of Cell Growth Phase, Salt Concentration, pH, and Binding Temperature on CAD4a Anchoring

The effect of cell growth phase on CAD4a binding capacity was investigated with frozen *L. fermentum* at mid-log and stationary phase. The influence of salt concentration on CAD4a binding to *L. fermentum* was tested using phosphate buffer (pH 5) supplemented with NaCl to final concentrations of 0.05, 0.1, 0.15, 0.2, 0.3, 0.4 M NaCl. The influence of pH was evaluated using PBS at pH 4.5, 5, 5.5, 6, 6.5, 7, 8, and 9. The effect of binding temperature was tested at 25, 30, and 37°C, with half-hourly timepoints up to 3 h. Protein concentration used was 2 μM in these studies; protein binding and fluorescence measurement were carried out as described above.

### Effect of Cell Pre-Treatment on CAD4a Anchoring

Frozen *L. fermentum* aliquots were treated with either 5 M LiCl or 10% v/v trichloroacetic acid (TCA) at 37°C with shaking for 1 h. Cells were washed twice with pH 7 PBS and once with pH 5 PBS before binding experiments.

### Binding Capacity of CAD4a on *L. fermentum*

Fresh overnight cultures of *L. fermentum* were washed twice with binding buffer (pH 5 PBS), diluted to OD_600_ = 1.5, and incubated with 70 μl of various concentrations of Sirius-CAD4a12 (0, 0.5, 1, 2, 3, 4, 5 μM). This was used to set up a standard curve to correlate RFU to protein concentration in the presence of cells. Subsequently, cell mixtures were pelleted and cell fluorescence determined as described above. All data points represent the average of at least three experiments. Non-linear regression analysis was used to fit the binding data to the Langmuir adsorption model to determine *B*_max_, the protein concentration at saturation. Assuming that the distribution of the anchored protein was uniform across the entire cell population, we calculated the average binding capacity per cell using *B*_max_ and the standard curve.

### Influence of Spacer Length on Activity of Surface-Displayed Superoxide Dismutase

Superoxide dismutase (SOD) from *Potentilla atrosanguinea* (Kumar et al., 2012) was engineered with C-terminal CAD4a and three different spacer lengths (12-, 24-, and 36-residues) between the enzyme and anchoring domain. A flexible (GGSG)_*x*_ spacer was used, where *x* = 3 for the 12-residue spacer, *x* = 6 for the 24-residue spacer, and *x* = 9 for the 36-residue spacer. Proteins were expressed in *E. coli*, and protein binding was performed with frozen log-phase *L. fermentum* as described above. After washing, cells were resuspended in binding buffer for the SOD activity assay.

### Superoxide Dismutase Activity Assay

SOD activity assay was done using a commercial SOD kit (Sigma-Aldrich 19160) according to the manufacturer's protocol. The average gradient over the first 10 min (linear range) from triplicates was used to calculate the activity for each sample.

### Cell Encapsulation

Cell encapsulation was adapted from a previously-described protocol (Nualkaekul et al., 2012). Low-viscosity alginate (Sigma-Aldrich) was prepared as a 6% w/v stock in distilled water. Low molecular weight chitosan (Sigma-Aldrich) was dissolved in 0.1 M acetic acid to a concentration of 0.5% w/v, and the final pH adjusted to 5. Fresh overnight cultures of *L. fermentum* were washed twice with binding buffer (pH 5 PBS), diluted to OD_600_ = 1.5, and incubated with 2 μM SOD-CAD4a12 or SOD-CAD4a36. After washing, 2 ml of cells was resuspended in 1.2 ml of 4% low-viscosity alginate (6% stock diluted with binding buffer) and mixed vigorously. The mixture was extruded dropwise into a 0.15 M CaCl_2_ bath (pH 5) using a 21G needle, and the beads were left to stir at room temperature for 1 h, then rinsed once with binding buffer. The beads were subsequently added to 0.5% chitosan and left to stir for 10 min, then washed twice with binding buffer and kept at 4°C till use. Empty beads and beads containing 1 μM SOD (without cells) were prepared as controls.

### *In vitro* Digestion

Simulated gastric fluid (SGF) was modified from Minekus et al. (2014) and prepared as a 1× concentrate. This consisted of: 6.9 mM KCl, 0.9 mM KH_2_PO_4_, 25 mM NaHCO_3_, 47.2 mM NaCl, 0.1 mM MgCl_2_(H_2_O)_6_, and 0.5 mM (NH_4_)_2_CO_3_. Pepsin (Sigma-Aldrich P6887) and all necessary chemicals were purchased from Sigma-Aldrich. Sodium citrate was dissolved in PBS (pH 5) at a concentration of 0.15 M. Ten alginate-chitosan beads were used for each condition tested. Beads were suspended in 125 μl distilled water and equivolume SGF, with addition of CaCl_2_ (final concentration 0.075 mM) and pepsin (final concentration 0.5 mg/ml). The mixture was incubated with shaking at 37°C for 2 hr, then rinsed twice with PBS (pH 7). Beads were incubated with pH 5 PBS as a control. The beads were dispersed in 250 μl citrate buffer held at 40°C, then centrifuged at 10,000 g for 10 min. The cell pellet was resuspended in 250 μl 1 × PBS (pH 5) for the SOD assay. Residual activity represents enzyme activity of SGF-treated beads relative to the control (beads in PBS).

### Statistical Analyses

Statistical analyses were performed using GraphPad Prism v8.0.1 for Windows. Two-tailed Student's *t*-tests were performed to determine the significance of differences in the binding studies. For curve fitting to the Langmuir model, non-linear regression analysis was performed assuming single-site saturation binding. *B*_max_ from the analysis was taken as the saturation RFU.

## Results

### Isolation of a Putative Cell Anchoring Domain (CAD4a) From *L. plantarum*

**Figure 1:**
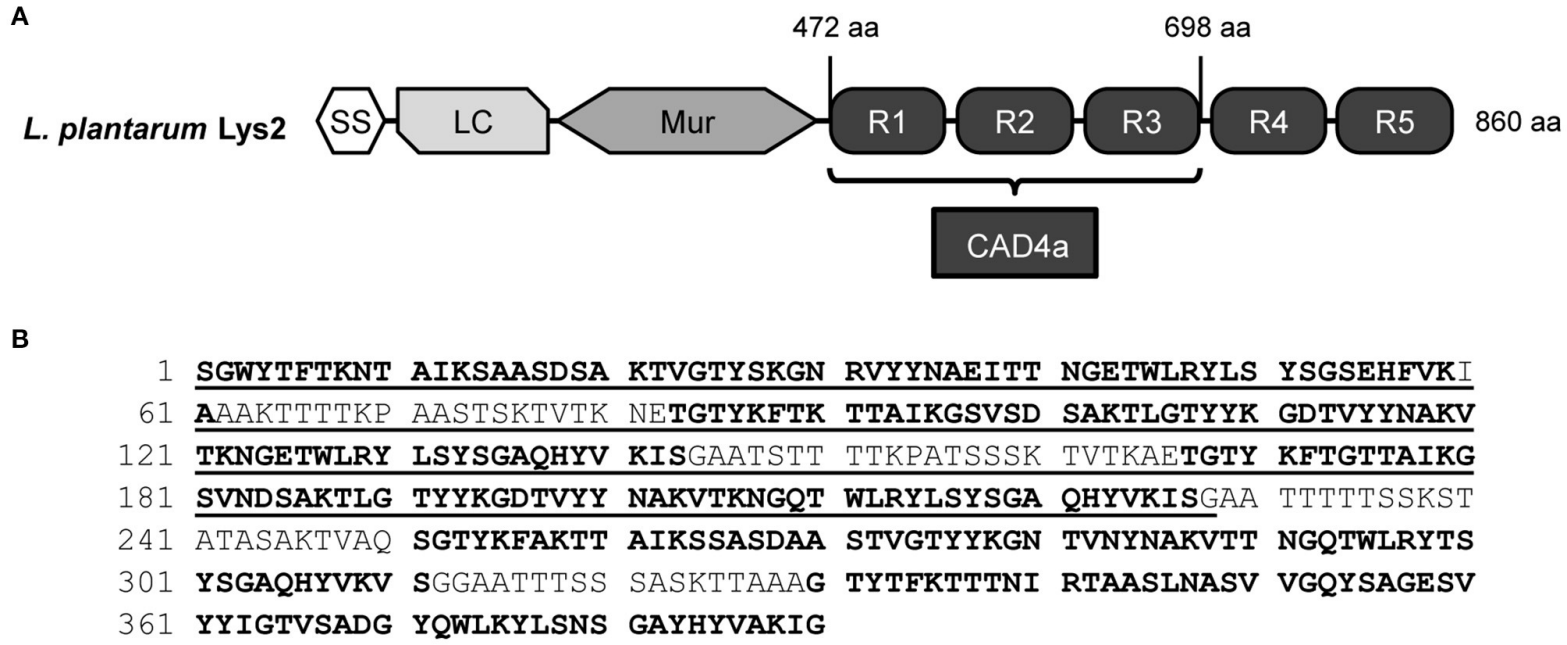
**(A)** Molecular organization of *L. plantarum* Lys2 with the C-terminal anchoring region. SS, signal sequence; LC, low complexity region; Mur, muramidase; R1–R5, SH3_5 repeats. **(B)** Amino acid sequence of the Lys2 anchoring region. Individual SH3_5 repeats are in bold, and the CAD4a domain has been underlined.

A search of the Pfam database revealed 83 homologs of the bacterial SH3 type 5 motif (SH3_5, Pfam PF08460) across the *Lactobacillus* and *Lactococcus* genera and their phages. This study focused on a previously uncharacterized SH3_5 anchoring domain in Lys2 (GenBank CCC80137), a muramidase from *L. plantarum* WCFS1. The full-length muramidase is an 860-amino acid protein with an expected molecular weight of 84 kDa, with five SH3_5 repeats (R1–5) at its C-terminus spanning residues 471 and 860 (Figure 1). Each repeat in Lys2 contains 61 residues, and the similarity between the five repeats vary from 50% to over 90%. The SH3_5 anchoring domain of Lys2 has 54% identity to that of Acm2 (GenBank CCC79778), another *L. plantarum* autolysin with similar molecular organization (Rolain et al., 2012). Comparing the first SH3_5 repeat of Lys2 to SH3_5 motifs in the proteins of *Streptococcus* (GenBank EQC72385, four SH3_5 repeats), *Staphylococcus* (GenBank AAB53783, single SH3_5), *L. lactis* phage 1358 (NCBI YP_009140409, single SH3_5), *Lactobacillus* phage ATCC 8014-B2 (NCBI YP_009783998, single SH3_5), and *Staphylococcus* phage K (GenBank AHB79986) showed identities of 40, 22, 27, 31, and 21%, respectively, thus the lactobacillal domain is homologous to but quite distinct from other SH3_5 domains in the group of Firmicutes (Supplementary Figure 1).

**Figure 2:**
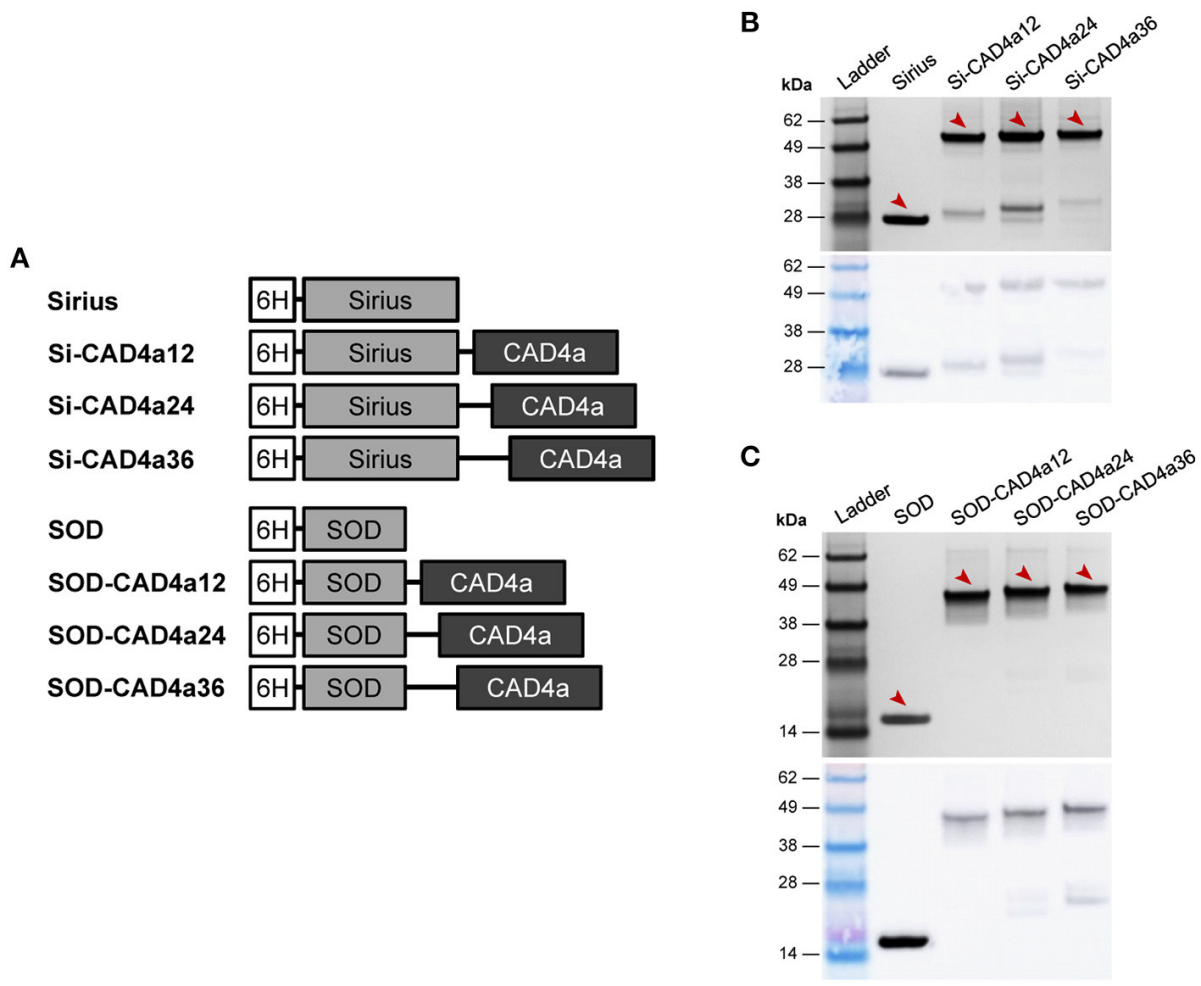
**(A)** Fusion protein constructs for surface display. 6H, His-tag; CAD4a, SH3_5 anchoring domain containing repeats R1–R3 (25 kDa); Si, Sirius blue fluorescent protein (28 kDa); SOD, superoxide dismutase from *Potentilla atrosanguinea* (17 kDa). Coomassie blue-stained SDS-PAGE gel (top) and western blot (bottom) of **(B)** Sirius and Si-CAD4a conjugates, and **(C)** SOD and SOD-CAD4a conjugates, all purified from *E. coli* cultures. Blots were labeled with anti-His antibody conjugated to HRP. Arrows indicate expected band position of the full-length proteins.

To assess the surface display potential of this five-repeat SH3_5 domain, we used the Sirius blue fluorescent protein as a reporter (Tomosugi et al., 2009). Sirius was chosen for its photostability at low pH (pKa < 3). The five-repeat domain was cloned downstream of Sirius in the pET22b plasmid, but the fusion protein was insoluble and difficult to purify (data not shown). A truncated domain containing only R1–R3 (henceforth called CAD4a) gave more tractable expression, and the Sirius conjugate (Si-CAD4a) was easily detected by SDS-PAGE and Western blot against the N-terminal His-tag (Figure 2B). There was some protein degradation during expression, but the bulk of the soluble fraction was the full-length protein.

### CAD4a Anchoring to LAB

**Figure 3:**
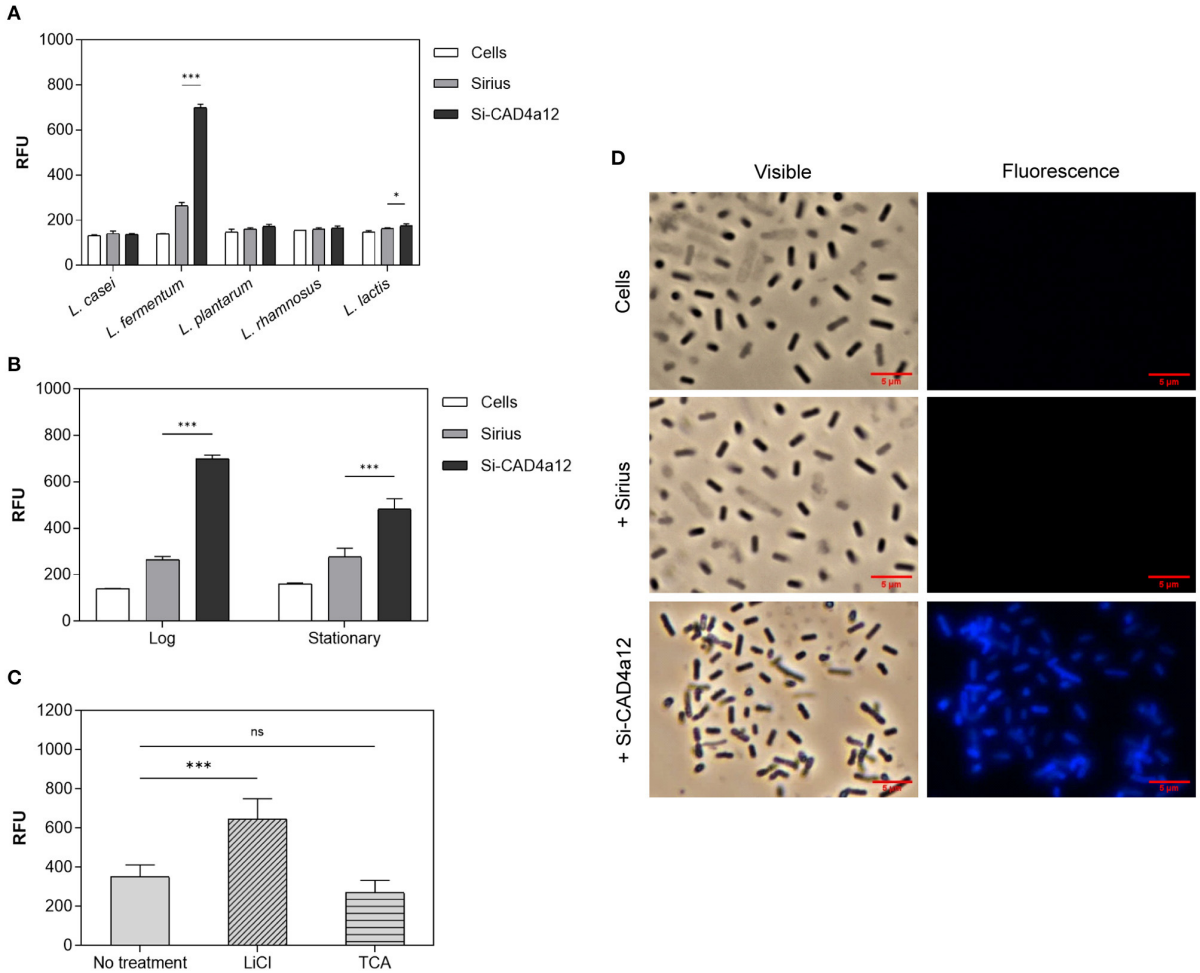
CAD4a12 anchors selectively to *L. fermentum*. Cell-associated fluorescence after Si-CAD4a12 was exposed to **(A)** *L. casei, L. fermentum, L. plantarum, L. rhamnosus*, and *L. lactis* in log-phase growth; and **(B)** *L. fermentum* in log- and stationary-phase growth. **(C)** Effect of pre-treatment of *L. fermentum* with 5 M LiCl or 10% v/v TCA on CAD-4a12 anchoring. **(D)** Fluorescence micrographs of log-phase *L. fermentum* (top), and cells exposed to Sirius (middle) and Si-CAD4a12 (bottom). Phase contrast image on left and fluorescence image on right (DAPI filter). *n* = 3. *ns, p* > 0.05; *p ≤ 0.05; ***p ≤ 0.001 vs. control.

Heterologous binding of Si-CAD4a12 (with a 12-residue spacer between Sirius and CAD4a) to LAB was tested using *L. lactis, L. casei, L. fermentum, L. plantarum*, and *L. rhamnosus* (Table 1). Bacteria in log-phase growth were incubated with 2 μM protein without shaking for 2 h at 37°C. As shown in Figure 3A, there was >2-fold increase in cell-associated fluorescence on *L. fermentum* after exposure to Si-CAD4a12, compared to Sirius without the anchoring domain. Fluorescence microscopy showed that the protein was displayed uniformly across the cell surface (Figure 3D). Negligible binding was seen with *L. lactis, L. casei, L. plantarum*, and *L. rhamnosus*, suggesting that the interaction between CAD4a and *L. fermentum* was selective. There was also more binding to log-phase compared to stationary-phase *L. fermentum* (Figure 3B). This could be due to actively dividing cells having more exposed cell wall structure and fewer surface proteins inhibiting access to the cell wall. Except where otherwise stated, all subsequent binding experiments were carried out with log-phase bacteria.

### Cell Wall Target of CAD4a

The SH3_5 domain is known to bind cross-linked peptidoglycan (PGN) (Desvaux et al., 2018). To confirm that CAD4a binds PGN, *L. fermentum* was pre-treated with either 5 M LiCl or 10% v/v TCA before exposure to Si-CAD4a12. TCA hydrolyzes teichoic acids (TAs), one of the major cell wall components, whereas 5 M LiCl removes non-covalently bound surface proteins. As shown in Figure 3C, treatment with LiCl led to a 2-fold increase in cell anchoring, likely because the removal of surface proteins exposed more binding sites within the cell wall. TCA treatment did not significantly impact CAD4a binding, thus the anchoring domain was not targeting cell wall TAs. Taken together, these results suggest that CAD4a is binding to PGN, the other major component of the Gram-positive cell wall. Further, since TCA treatment significantly reduced cell viability (results not shown) but not CAD4a anchoring, changes to cell viability are not likely to impact CAD4a-mediated protein display, so long as the PGN matrix remains intact.

### Factors Influencing Binding of CAD4a to *L. fermentum*

**Figure 4:**
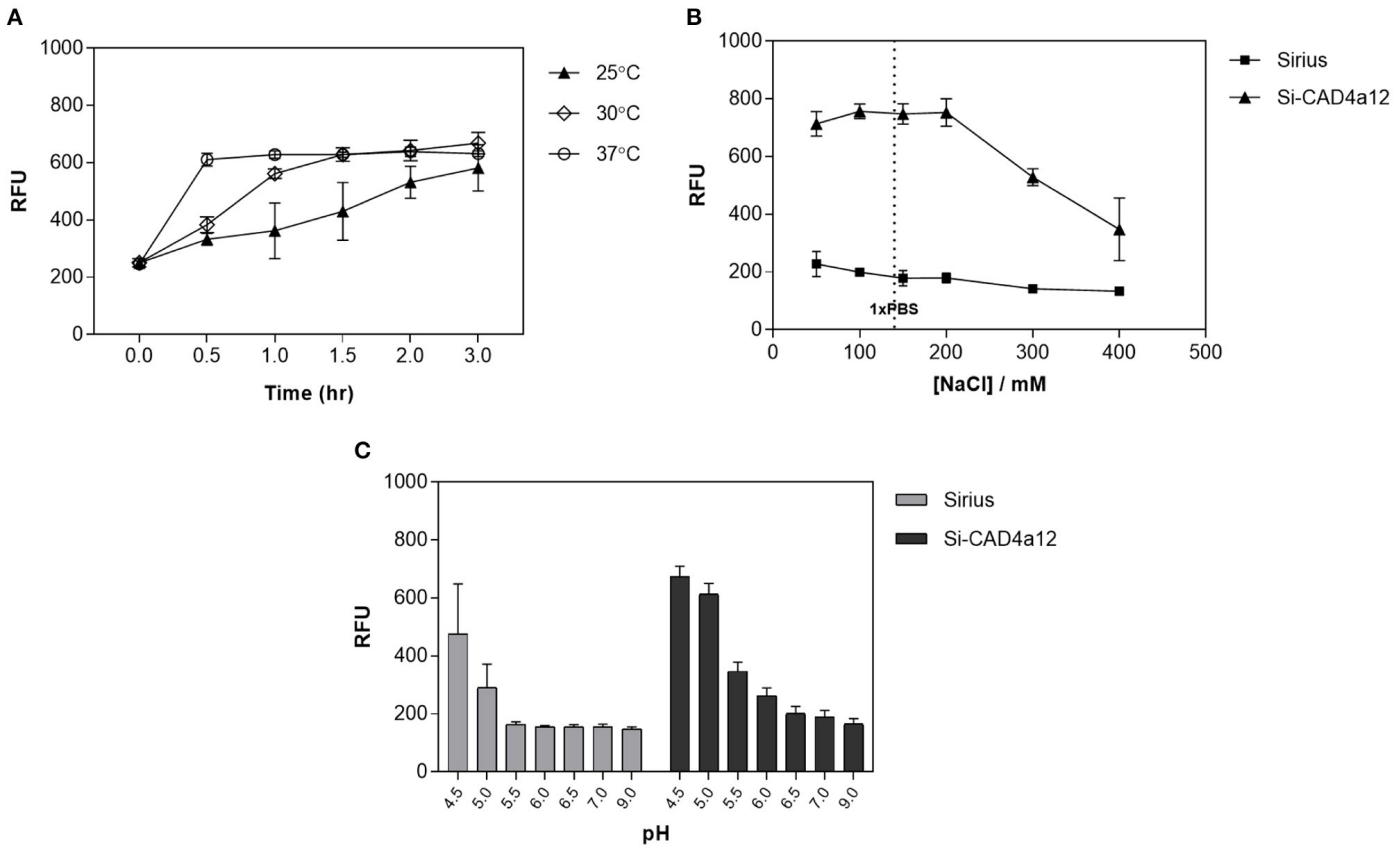
Anchoring of Si-CAD4a12 under different conditions. **(A)** Time-course of anchoring at different temperatures. Anchoring at **(B)** different NaCl concentrations, and **(C)** different pH. *n* = 3.

We investigated several binding conditions to see if we could improve surface display on *L. fermentum*. Ambient temperature had an effect on the rate of binding, with noticeably slower binding at 25°C compared to 37°C (Figure 4A). At 30 and 37°C, maximum binding was achieved within 1.5 h. Ionic strength had a negligible effect on binding at low and physiological salt concentrations (≤ 150 mM NaCl), but high salt concentrations (> 200 mM NaCl) adversely affected anchoring, possibly by disrupting the non-covalent interactions between CAD4a and PGN (Figure 4B). We found that pH 5 was optimal for CAD4a anchoring; there was no binding above pH 6, whereas below pH 5, non-specific binding of Sirius became significant and the contribution of CAD4a was less clear (Figure 4C).

**Figure 5:**
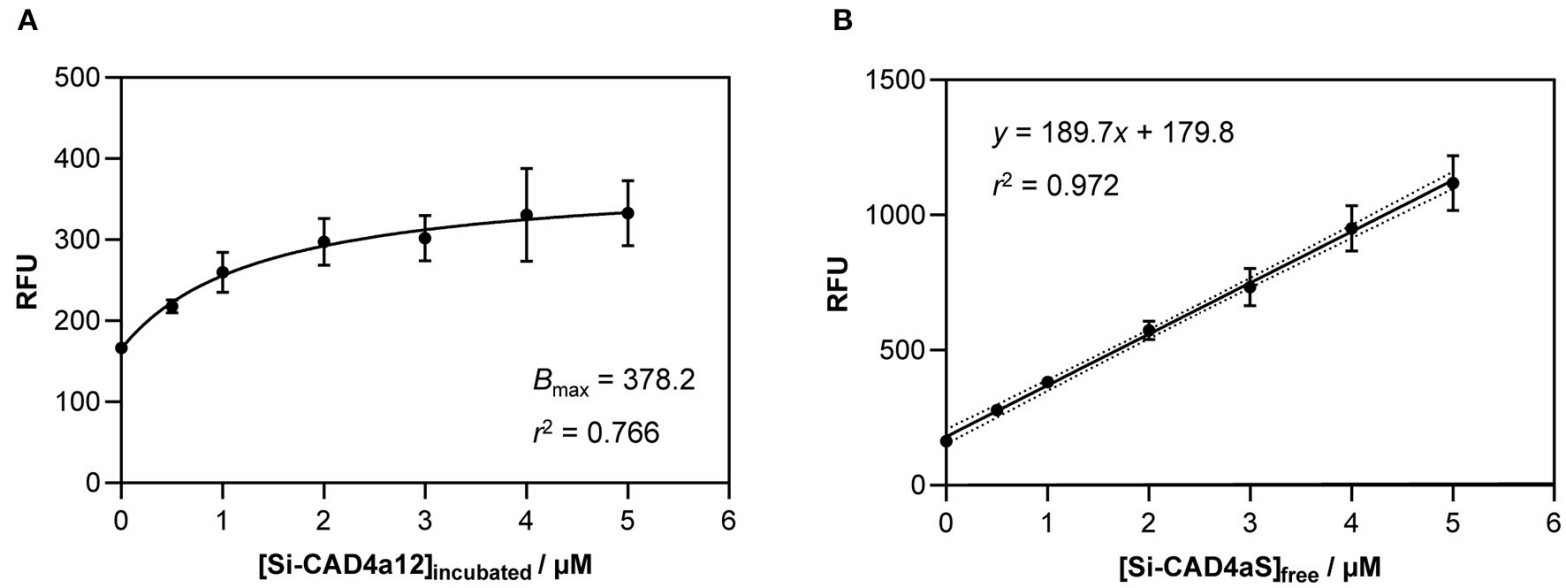
Binding curve for anchoring of Si-CAD4a12 to overnight cultures of *L. fermentum*. Data points represent the average and standard deviation of at least three independent experiments. Points were fitted to a Langmuir adsorption isotherm to determine fluorescence at saturation **(A)**, and concentration at saturation was calculated from a standard curve **(B)**.

To determine the binding capacity of CAD4a on stationary-phase *L. fermentum*, different concentrations of Si-CAD4a were mixed with overnight cultures diluted to OD_600_ = 1.5 (~10^9^ cells/ml). As shown in Figure 5A, cell-associated fluorescence increased in a dose-dependent manner before reaching a plateau, with *B*_max_ determined to be ~378 RFU after fitting to a Langmuir adsorption isotherm (*r*^2^ = 0.766). This corresponded to a saturation concentration of 1.05 μM protein (Figure 5B), or an average of 5 × 10^5^ molecules per cell.

### Heterologous Display of Superoxide Dismutase via CAD4a

To demonstrate the surface display of a functional protein using CAD4a, we cloned and expressed a superoxide dismutase (SOD) from *P. atrosanguinea* with CAD4a at its C-terminus. SOD is an enzyme that scavenges reactive oxygen radicals and has potential application in treating intestinal inflammation, based on positive outcomes in mouse models (Seguí et al., 2004). We constructed three SOD-CAD4a variants with 12-, 24-, and 36-residue glycine-serine (GS) spacers (Figure 2A) to examine if spacer length affects protein activity after anchoring. Active SOD is a homodimeric complex, and a short linker could have impacted its dimerization and thus enzyme activity. Full-length protein was obtained for all three variants following expression and purification from *E. coli* cultures (Figure 2C). A comparison of enzyme activity showed that SOD-CAD4a12 had slightly reduced activity compared to SOD and the other spacer variants, although the difference was not statistically significant (Supplementary Figure 2A). We did not observe the same effect of spacer length on the monomeric Sirius, as fluorescence output of Sirius conjugates with different spacer lengths did not differ significantly (Supplementary Figure 2B).

**Figure 6:**
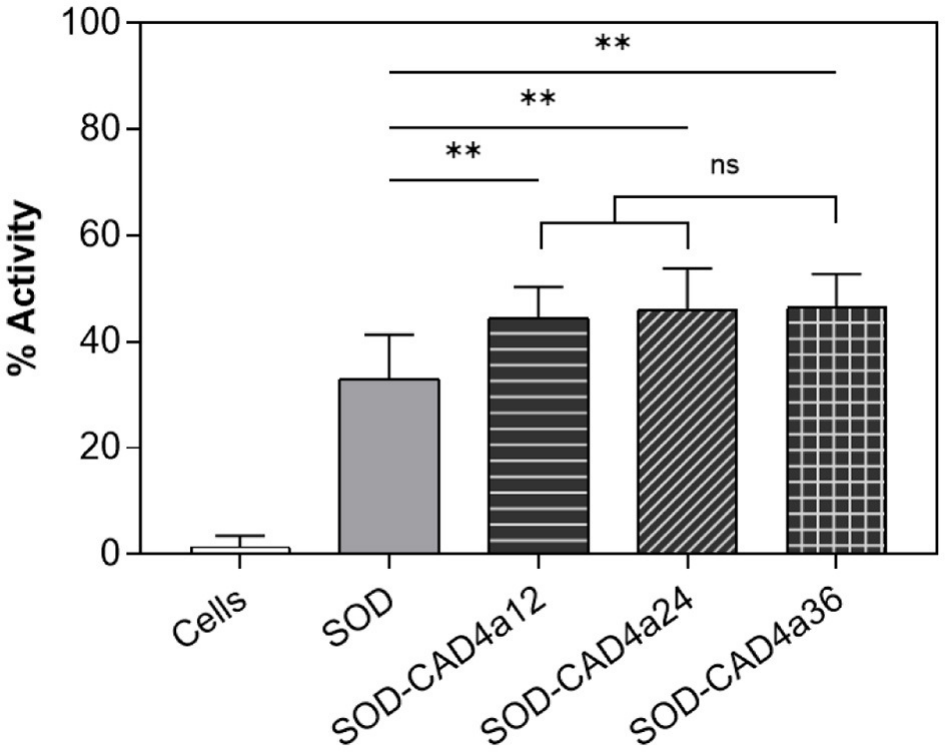
Cell-associated enzyme activity after exposure of SOD and SOD-CAD4a variants to log-phase *L. fermentum*. *n* = 3. *ns, p* > 0.05; **p ≤ 0.01 vs. control.

The SOD variants were used for subsequent binding experiments. As shown in Figure 6, there was more non-specific binding for SOD compared to Sirius, but the addition of the anchoring domain increased cell-associated enzyme activity by ~40%. All three spacer variants gave very similar activity after cell anchoring (Figure 6), thus spacer length had minimal effect on the functionality of the anchored domain. The native spacer in the *L. plantarum* muramidase is 33 amino acids long. Our results suggest that shorter spacers could be used, although the ideal length is protein-dependent, and should be optimized based on size and multimericity of the target protein.

### Gastric Resistance of Encapsulated SOD-Coated Bacteria

**Figure 7:**
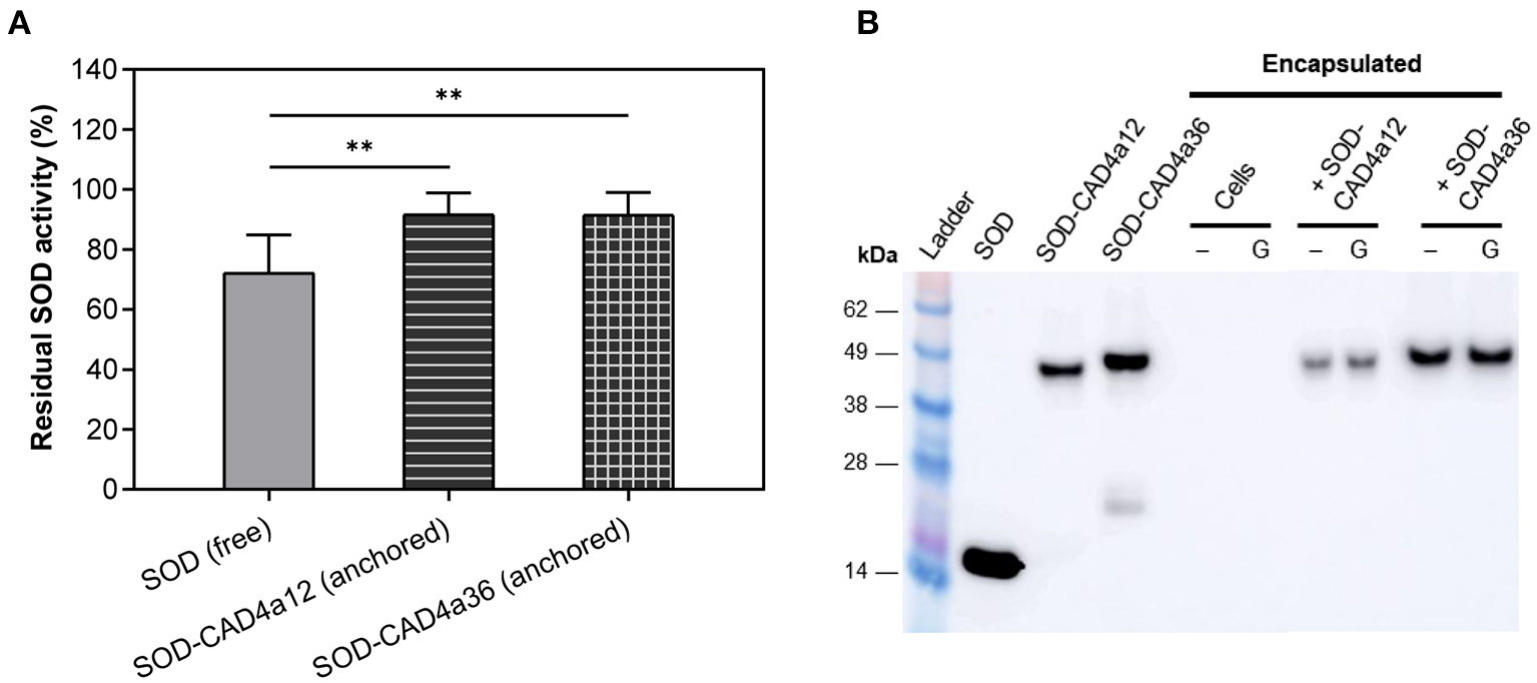
Outcome of *in vitro* gastric digestion of free SOD and SOD-coated *L. fermentum*, both in chitosan-alginate beads. Beads were digested with pH 3 SGF containing pepsin, then dispersed and the cells pelleted down for the SOD activity assay. **(A)** Residual enzyme activity, calculated as cell-associated activity after digestion relative to undigested control beads left in pH 5 PBS. **(B)** Western blot of resuspended cell pellets that were untreated (–) or underwent gastric digestion (G). Proteins were labeled with anti-His antibody. The first three lanes after the protein ladder are pure proteins for reference. **p ≤ 0.01 vs. control.

We found that anchored Si-CAD4a was sufficiently surface-exposed to be digested when cells underwent simulated gastric digestion (Supplementary Figure 3). There was thus a need to protect surface-displayed protein from detachment and/or degradation under changing and potentially adverse ambient conditions. Nualkaekul et al. showed that a matrix of alginate coated in chitosan helped to buffer pH changes and maintained the viability of *L. plantarum* in acidic media (Nualkaekul et al., 2012). To maintain optimal conditions for CAD4a anchoring during encapsulation, SOD-coated cells mixed with alginate were gelled in a Ca^2+^ bath at pH 5, and the alginate beads were then coated with a chitosan solution, also at pH 5. Gastric resistance was evaluated using simulated gastric fluid (SGF) at pH 3 containing the gastric protease pepsin (Minekus et al., 2014). Following gastric digestion, beads were treated with a citrate solution at pH 5 to chelate the Ca^2+^ ions and disperse the matrix. Subsequent activity assays on the pelleted cells showed that over 90% of cell-associated enzyme activity was retained (Figure 7A), thus the polymer matrix offered substantial protection for the surface-displayed enzyme. In comparison, there was less residual enzyme activity (72%) in beads that contained unanchored SOD (Figure 7A), suggesting that cell anchoring also confers some protection to the protein, perhaps by limiting protein diffusion within the matrix. A Western blot of the cell pellet showed that the anchoring domain was intact, thus the enzyme likely remained anchored to the cell surface (Figure 7B).

## Discussion

The first SH3_5 anchoring motif was identified in staphylococcal endolysins and helped to localize the latter to its cell wall targets (Baba and Schneewind, 1996; Gründling and Schneewind, 2006). These domains, along with similar ones from phages, contained a single SH3_5 motif. In contrast, SH3_5-based anchoring domains from *Streptococcus* and *Lactobacillus* can have multiple SH3_5 repeats, up to six in tandem for some species. Such domains from *Lactobacillus* have not been investigated for protein surface display, where the multiplicity of SH3_5 may provide stronger cell wall binding. This work examined the anchoring domain of the *L. plantarum* Lys2 autolysin, which contains five terminal SH3_5 repeats. From the Lys2 cell wall targeting region, a truncated three-repeat domain, which we named CAD4a, was successfully expressed as part of fusion proteins. We tested five strains of LAB for anchoring of CAD4a, and found selective binding to *L. fermentum*. It was surprising that CAD4a bound poorly to the *L. plantarum* strain that we tested (ATCC 8014), since it was derived from another *L. plantarum* strain (WCFS1). However, the surface of *L. plantarum* is known to be covered in capsular polysaccharides, which were shown to inhibit Acm2 binding in a previous study (Beaussart et al., 2013), and could have had a similar shielding effect toward CAD4a anchoring. Although the surface composition of *L. fermentum* is not well-characterized, it is likely that a lack of significant polysaccharide coverage made this bacterium a more ideal host for CAD4a binding. Alternatively, all five of the SH3_5 repeats might be essential for proper binding to the *L. plantarum* cell wall, similar to the LysM domain in lactococcal AcmA, which requires all three of its LysM repeats for optimal PGN binding (Steen et al., 2005).

We have shown that CAD4a binds to cell wall peptidoglycan (PGN), though its ligand is uncertain. Existing molecular models for PGN-SH3_5 interactions were based on staphylococcal domains, where it was shown that SH3_5 recognized pentapeptide cross-bridges in PGN (Mitkowski et al., 2019; Gonzalez-Delgado et al., 2020). However, such cross-bridges are absent in lactobacilli (Kleerebezem et al., 2010), and the section in staphylococcal SH3_5 that binds the cross-bridges is notably absent from CAD4a. Instead, CAD4a may anchor similar to LysM, another PGN-binding motif commonly used for surface display. LysM recognizes a generic GlcNAc-X-GlcNAc motif in PGN glycans, but its binding affinity is modulated by neighboring peptide stems, thus providing some measure of selectivity (Mesnage et al., 2014). It was recently shown that the SH3_5 domain of *L. plantarum* Acm2 autolysin also has an affinity for PGN glycan chains containing GlcNAc, although the authors did not examine the influence of PGN peptide stems (Beaussart et al., 2013). It is thus possible that the SH3_5 and LysM binding motifs recognize similar PGN ligands, and cannot be used concurrently for protein display.

Stationary-phase bacteria bound less protein than log-growth bacteria, but are more robust to handling and gastrointestinal transit, and are thus of greater practical importance, especially for *in vivo* delivery applications that require viable cells (Brashears and Gilliland, 1995; Lorca and De Valdez, 1999; Corcoran et al., 2004; Broeckx et al., 2020). Stationary-phase *L. fermentum* bound 10^5^ molecules of monomeric protein per cell. This compares well with previously reported binding capacities for LysM and S-layer-derived anchoring domains, which ranged from 10^3^ to 10^6^ molecules/cell (Bosma et al., 2006; Hu et al., 2010, 2011; Ravnikar et al., 2010). The binding capacity is likely to vary with the size and multimericity of the protein. Improvement in binding was reported for other anchoring domains after pre-treating bacteria with various solvents and detergents (Hu et al., 2010, 2011; Xu et al., 2011); we have also shown that 5 M LiCl treatment improves binding of CAD4a (Figure 3C). However, such treatments disrupt the proteosurfaceome of bacteria, and could have knock-on effects on cell viability and host-microbe interactions *in vivo*, and so should be used judiciously.

The optimal pH for CAD4a anchoring (pH 5) means that coated bacteria must be kept under slightly acidic conditions to maintain surface display. This precludes the display of acid-sensitive proteins—an important limitation of the CAD4a anchoring domain—although many lactobacilli can still thrive under these conditions, and *L. fermentum* is known to tolerate pH values down to 4.5 (Calderon Santoyo et al., 2003; LeBlanc et al., 2004). Encapsulation in a polymer matrix can further protect the anchored protein from the harsh conditions of gastrointestinal transit. We have shown that one such matrix, a composite of the natural polymers alginate and chitosan which can be assembled under acidic conditions, can protect surface-anchored protein from gastric digestion; other enteric coatings are reviewed elsewhere (Ramos et al., 2018; Gomand et al., 2019).

We have shown that CAD4a could be used to display SOD on the surface of *L. fermentum*, with minimal effect on enzyme functionality. Various strains of *L. fermentum* are known to have probiotic effects (Geier et al., 2007; Peran et al., 2007; Mikelsaar and Zilmer, 2009; Garcia-Castillo et al., 2019); whether these could enhance the therapeutic outcome of displayed functionalities could form the basis of future studies. It was shown previously that recombinant lactobacilli engineered to secrete SOD can ameliorate intestinal inflammation in mouse models of inflammatory bowel disease (Carroll et al., 2007; Watterlot et al., 2010; Hou et al., 2014). The SOD produced complemented inherent antioxidative and immunomodulatory properties of the probiotic *Lactobacillus* strains. However, such genetically modified bacteria might face stringent regulatory scrutiny and poor public acceptance. Heterologous display of therapeutic domains like SOD using CAD4a on wild-type LAB could get around these constraints, since no recombinant DNA is introduced to lactobacilli, and these bacteria could potentially be sourced from healthy microbiomes. We are currently looking into protein surface display using CAD4a for treatment of intestinal disease and for mucosal vaccination.

To conclude, we have characterized the use of CAD4a, a novel SH3_5-based anchoring domain, for surface display of heterologous proteins on LAB. C-terminal conjugation of the domain enabled the anchoring and display of two different proteins on *L. fermentum*, with optimal anchoring at pH 5, and up to 10^5^ monomeric protein displayed per cell. Besides the delivery of therapeutic proteins to the gut, we foresee that surface display mediated by CAD4a could be also be used in the development of mucosal vaccines and for industrial biocatalysis.

## Data Availability Statement

The original contributions presented in the study are included in the article/Supplementary Material, further inquiries can be directed to the corresponding author/s.

## Author Contributions

PT and DO conceptualized the study and interpreted the results. PT designed and performed the experiments. PL helped with cloning and performed experiments involving SOD. PT drafted the manuscript and DO edited it. All authors reviewed the final manuscript.

## Conflict of Interest

The authors declare that the research was conducted in the absence of any commercial or financial relationships that could be construed as a potential conflict of interest.
