# Supplementary material for: A SH3_5 Cell Anchoring Domain for Non-recombinant Surface Display on Lactic Acid Bacteria
Source: Front Bioeng Biotechnol. 2021 Jan 27;8:614498. doi: 10.3389/fbioe.2020.614498 (PMC7873443; doi:10.3389/fbioe.2020.614498)
Supplement: Supplementary file 1 [file Data_Sheet_1.docx]

Supplementary Material

**Supplementary Table 1.** Primers and synthetic gene fragments used in this study.

|  | **Sequence** |
| --- | --- |
| **Primers** |  |
| F1 | 5’−tgagatccggctgctaacaaagcccgaaag |
| F15 | 5’−GGCGGGTCCGGCGGTGGAAGTGGCGGTGGTAGTGGCGGAGGTTCGGGGGGTGGCAGCG |
| F18 | 5’−ccacggtgggtctggggggggatccggcATGGCAAAGGGAGTGGCGGTACTGT |
| R1 | 5’−GCCTTGGTTTTCTAATTTTGGTTCAAAGAAAGCT |
| R4 | 5’−GCCGGATCCCCCCCCAGACCCAC |
| R5 | 5’−ctttcgggctttgttagcagccggatctca |
| R6 | 5’−GCCTGACCCACCACCACTCCCGCCGCCGGAACCGCCTCCTGATCCACCTCCGCTGCCACCCCCCGAACCTCCcttgtacagctcgtccatgccgagagtg |
| R7 | 5’−GCCTGACCCACCACCACTCCCGCCGCCGGAACCGCCcttgtacagctcgtccatgccgagagtg |
| R24 | 5’−GACCCACCACCACTCCCGCCGCCGGAACCGCCTCCTTGAAGGCCAATGATGCCAC |
| R25 | 5’−CGCTGCCACCCCCCGAACCTCCTCCTTGAAGGCCAATGATGCCAC |
| R26 | 5’−GCCACTACCACCGCCACTTCCACCGCCGGACCCGCCcttgtacagctcgtccatgccgagagtg |
| R27 | 5’−GCCACTACCACCGCCACTTCCACCGCCGGACCCGCCTCCTTGAAGGCCAATGATGCCAC |
| R37 | 5’−ctttcgggctttgttagcagccggatctcaTCCTTGAAGGCCAATGATGCCACATGC |
| **Gene fragments** |  |
| G5 (Sirius) | GTGGGTCTGGGGGGGGATCCGGCgtgagcaagggcgaggagctgttcaccggggtggtgcccatcctggtcgagctggacggcgacgtaaacggccacaggttcagcgtgtccggcgagggcgagggcgatgccacctacggcaagctgaccctgaagctcatctgcaccaccggcaagctgcccgtgccctggcccaccctcgtgaccaccctgcaattcggcgtgctgtgcttcgcccgctaccccgaccacatgaagcagcacgacttcttcaagtccgccatgcccgaaggctacgtccaggagcgtaccatcttcttcaaggacgacggcaactacaagacccgcgccgaggtgaagttcgagggcgacaccctggtgaaccgcatcgagctgaagggcatcgacttcaaggaggacggcaacatcctggggcacaagctggagtacaacgggataagctcaaacgtatatatcaccgccgacaagcagaagaacggcatcaaggcccacttcaagatccgccacaacatcgaggacggcggcgtgcagctcgccgaccactaccagcagaacacccccatcggcgacggccccgtgctgctgcccgacaaccactacctgagcgtccagtccaagctgagcaaagaccccaacgagaagcgcgatcacatggtcctgctggagtccgtgaccgccgccgggatcactctcggcatggacgagctgtacaagtgagatccggctgctaacaaagcccgaaag |
| G10 (CAD4a) | CGGGAGTGGTGGTGGGTCAGGCTCTGGCTGGTACACTTTTACGAAAAATACCGCCATTAAATCCGCTGCCTCTGACTCCGCTAAAACCGTGGGTACATATTCAAAGGGAAATCGTGTTTATTACAATGCGGAGATTACAACTAACGGCGAGACATGGCTTCGCTACCTTAGCTATTCGGGCTCTGAACACTTCGTGAAGATTGCGGCTGCTAAAACTACAACCACAAAGCCAGCTGCATCTACCTCTAAAACTGTTACCAAGAACGAGACAGGAACTTATAAATTCACCAAAACCACGGCCATCAAGGGCTCGGTTTCGGATTCCGCCAAGACCCTGGGAACTTACTACAAAGGGGACACCGTATATTATAATGCCAAAGTTACTAAAAATGGAGAGACGTGGCTTCGTTACCTGTCGTATTCCGGCGCTCAACACTATGTGAAGATTTCTGGCGCAGCCACGTCGACTACCACGACAAAACCCGCAACGTCTTCGAGTAAGACCGTCACGAAAGCTGAGACTGGCACATATAAATTTACAGGCACTACGGCTATTAAGGGCAGCGTGAACGATTCGGCCAAAACCTTAGGAACATACTATAAGGGGGACACCGTTTATTACAATGCGAAAGTTACAAAAAATGGGCAGACCTGGTTACGCTATTTGTCGTATTCCGGGGCACAGCACTACGTGAAAATCTCTGGTtgagatccggctgctaacaaagcccgaaag |
| G29 (SOD) | ATGGCAAAGGGAGTGGCGGTACTGTCGTCCAGTGAAGGCGTCGCTGGGACGATACTTTTTACACAGGAAGGTGATGGACCGACAACTGTCACTGGGAACATTAGTGGACTGAAACCGGGATTACATGGGTTCCACGTCCATGCCCTTGGCGACACCACCAACGGATGTATGTCAACAGGACCACATTTCAATCCGGCGGGCAAAGAACATGGGTCCCCTGAGGACGAAACCCGCCATGCGGGCGACTTGGGTAACATCACGGTGGGAGATGACGGAACGGCGTGTTTTACTATCGTAGATAAACAGATTCCTTTAACCGGACCGCACTCAATTATCGGTCGGGCCGTCGTGGTGCACGCCGACCCGGACGACTTAGGCAAGGGGGGACACGAGCTTTCAAAATCTACGGGAAATGCTGGAGGTCGGATCGCATGTGGCATCATTGGCCTTCAAGGA |


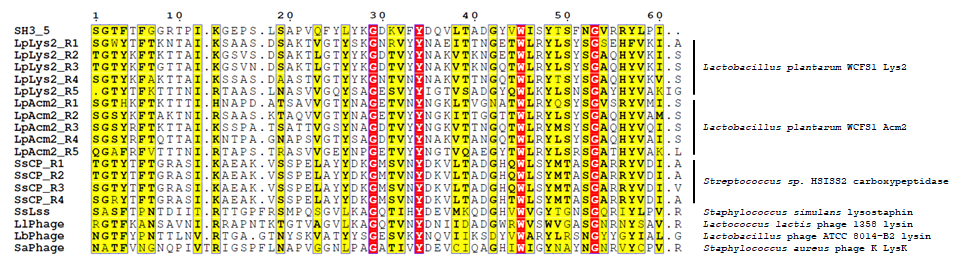
**Supplementary Fig. 1.** Comparison of SH3_5 sequences from proteins of various Firmicutes and their phages. The SH3_5 consensus sequence (first row) was obtained from the NCBI Protein database. The remaining sequences were obtained from the Pfam database. Multiple sequence alignment was carried out using the M-Coffee web server ([Moretti et al.](file:///C:\Users\tayrpk\Dropbox\Front%20Bioeng%20Biotechnol%20Paper%20(CAD4a)\Supplementary%20Material.docx#_ENREF_1), 2007) and results were further processed for visualization on the ESPript 3.0 web server ([Robert and Gouet, 2014](file:///C:\Users\tayrpk\Dropbox\Front%20Bioeng%20Biotechnol%20Paper%20(CAD4a)\Supplementary%20Material.docx#_ENREF_2)). **Identical** residues are in white on a framed red background. Residues with **similar physico-chemical properties** are in bold on a framed yellow background.

**
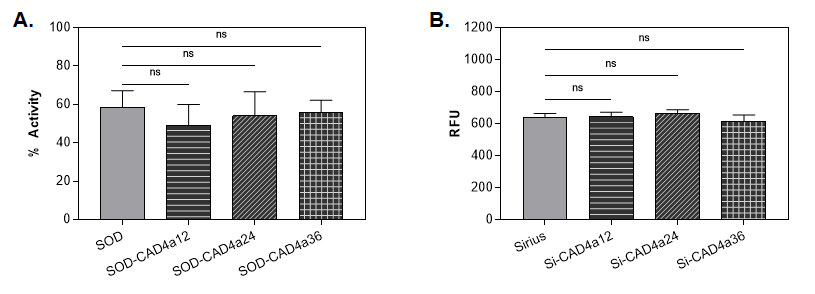
**

**Supplementary Fig. 2.** **(A)** Enzyme activity of 2 μM SOD compared to SOD-CAD4a spacer variants. **(B)** Fluorescence output of 2 μM Sirius compared to Si-CAD4a spacer variants. Proteins were diluted in pH 7.4 PBS for the activity or fluorescence assay. *n* = 3. *ns*, *p* > 0.05.

**
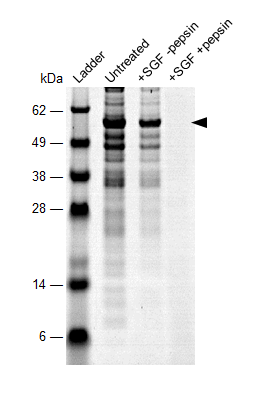
**

**Supplementary Fig. 3.** Gastric resistance of cells coated with Si-CAD4a12. Coated cells were resuspended in pH 5 PBS (control, Lane 2) or SGF with (Lane 4) or without pepsin (Lane 3), and left to shake for 1 hr at 37°C. The cells were then pelleted, washed once with pH 5 PBS, lysed at 95°C for 10 min, and run on an SDS-PAGE gel. Gels were stained with Coomassie Blue for imaging. Expected molecular weight of Si-CAD4a12 is indicated by the black arrow. The displayed protein was digested by pepsin, suggesting that cell anchoring alone does not provide sufficient protection against adverse ambient conditions.

**References**

Moretti, S., Armougom, F., Wallace, I. M., Higgins, D. G., Jongeneel, C. V., and Notredame, C. (2007). The M-Coffee web server: a meta-method for computing multiple sequence alignments by combining alternative alignment methods. *Nuc. Acids Res.* 35 (Web Server issue), W645–W648. doi: 10.1093/nar/gkm333

Robert, Xavier and Gouet, Patrice (2014), 'Deciphering key features in protein structures with the new ENDscript server', *Nuc. Acids Res.* 42 (W1), W320-W24. doi: 10.1093/nar/gku316
